# First report on *bla*_NDM-1_-producing *Acinetobacter baumannii* in three clinical isolates from Ethiopia

**DOI:** 10.1186/s12879-017-2289-9

**Published:** 2017-03-01

**Authors:** Michael Pritsch, Ahmed Zeynudin, Maxim Messerer, Simon Baumer, Gabriele Liegl, Soeren Schubert, Thomas Löscher, Michael Hoelscher, Tefara Belachew, Andrea Rachow, Andreas Wieser

**Affiliations:** 1 Division of Infectious Diseases and Tropical Medicine, Medical Center of the University of Munich (LMU), Leopoldstr. 5, 80802 Munich, Germany; 2 German Center for Infection Research (DZIF), partner site Munich, Munich, Germany; 3 College of Health Sciences, Jimma University, Jimma, Ethiopia; 4 CIH LMU Center for International Health, Ludwig-Maximilians-Universität (LMU), Munich, Germany; 5 Department of Bacteriology, Max von Pettenkofer-Institute (LMU), Munich, Germany; 6 Faculty of Biology, Ludwig-Maximilians-Universität (LMU), Planegg-Martinsried, Germany

**Keywords:** NDM-1, New Delhi metallo-beta-lactamase, *Acinetobacter baumannii*, Carbapenemase, Ethiopia, East-Africa

## Abstract

**Background:**

Multidrug-resistant Gram-negative bacterial infections are recognized as one of the major threats to global health. In this study, we describe for the first time *bla*_NDM-1_ gene carrying organisms from Ethiopia consisting of three *Acinetobacter baumannii* isolates from patients in Jimma.

**Methods:**

Besides phenotypic antimicrobial susceptibility testing, molecular strain typing and sequencing was performed to describe the phylogenetic relation of the Ethiopian isolates in detail in relation to published isolates from all over the globe.

**Results and discussion:**

Three multi-resistant, *bla*_NDM-1_-positive *Acinetobacter baumannii* isolates, most likely a local clonal diffusion, were isolated. Two of the three isolates described within this study were untreatable with the locally available antimicrobials and were only susceptible to polymyxin B and amikacin. The genome sequences confirmed the isolates to be distinct from the outbreak strains reported from Kenya, the only other characterized *bla*_NDM-1_ positive *Acinetobacter baumannii* strains in East Africa so far. Up to date, no other bacterial species were found to harbour the gene cassette in Jimma and conjugation to *E. coli* was not successful under laboratory conditions. However, natural transmission to other bacteria seems likely, given the evident lack of hygienic precautions due to limited resource settings.

**Conclusions:**

The detected isolates could solely be the tip of the iceberg regarding the presence of NDM-1 producing organisms in the region, as only a limited number of bacterial isolates were evaluated so far and until recently, susceptibility testing and isolation of bacteria could hardly be performed in clinical patient care. These multi-drug resistant organisms pose a serious threat to antimicrobial treatments in Jimma, Ethiopia.

**Electronic supplementary material:**

The online version of this article (doi:10.1186/s12879-017-2289-9) contains supplementary material, which is available to authorized users.

## Background

Multidrug-resistant Gram-negative bacterial infections are recognized as one of the major threats to global health, the leading cause of nosocomial infections both in industrialized as well as developing countries and leading to a high economic burden [1–4]. These organisms have efficient capabilities to accumulate resistant traits and pass the genetic determinants to other bacteria in order to allow them to become drug-resistant as well [2, 5]. Apart from the genetic characteristics, other pharmacological and societal factors including human behaviour at many levels play a significant role for the emergence and spread of bacterial pathogens resistant to antimicrobials [6, 7]. In developing countries such as Ethiopia, where there is a high burden of infectious diseases combined with a lack of resources, the impact on society is devastating [3, 8]. The sky-rocketing rates of antibiotic resistant bacteria in developing countries are a big threat to the whole health care system including the patients in the respective areas as well as at the global level [2, 3]. The ever increasing travel of people around the globe is facilitating and speeding up the spread of resistant organisms across all borders [9, 10]. Although we have learned a lot about the genetics and molecular biology of multidrug-resistant bacteria from investigations in industrialized countries, it is in developing countries where more studies are needed and are a critical public health priority in order to help with the design of containment strategies [2, 3].

One of the most common and feared multidrug-resistant organisms responsible for hospital-acquired infections are *Acinetobacter baumannii* producing either extended-spectrum ß-lactamases (ESBL), carbapenemases or a combination of different factors [11]. Strains of *A. baumannii* resistant to carbapenems (CRAB) are regarded as sentinels for emerging antimicrobial resistance [12]. The most frequent mechanism of resistance are carbapenem-hydrolyzing class D ß-lactamases (CHDL) followed by class B metallo-ß-lactamases (MBL) [13, 14]. So far, there are very few reports on CRAB in Africa, and those published are mostly from northern or southern African countries, which leaves the precise molecular epidemiology of those pathogens on this continent unidentified [13, 15]. In the current literature on CRAB from African countries, multiple CHDL (OXA-23, OXA-24/40, OXA-58, and OXA-97) and some MBL (such as NDM-1) have been described [13, 15, 16].

Jimma University Specialized Hospital (JUSH) is located in southwest Ethiopia and constitutes the only referral hospital for over 15 million people in the catchment area [17]. Until 2013, no susceptibility testing and isolation of bacteria could be performed for clinical patient care at JUSH. As a result, antibiotic therapy was performed only empirically, predominantly with combinations of different antimicrobial substances. The overuse of antimicrobial drugs for treatment and prophylaxis most likely serves as one of the key drivers of resistance development and increasing treatment failure rates in the patients [3, 17]. In a collaboration of the University of Munich (LMU) and the German Center for Infection Research from Germany (DZIF) together with JUSH, the establishment of a bacteriology laboratory including an infectious diseases consulting service was initiated. Since late 2013, patient samples can be investigated using common culture based techniques and phenotypic resistance testing up to the standards for patient care.

This report describes to our knowledge the first analysis of NDM-1 producing *Acinetobacter baumannii* isolates in Ethiopia, which were obtained from patient samples in JUSH. Besides phenotypic antimicrobial susceptibility testing, molecular strain typing and sequencing was performed to describe the phylogenetic relation of the Ethiopian isolates in relation to published isolates from all over the globe.

## Methods

### Study setting, specimen collection and patient characteristics

Jimma University Specialized Hospital (JUSH) is located in Jimma city, Oromiya regional state, situated at a distance of more than 350 km southwest of the Ethiopian capital city Addis Ababa. At the time of the study, JUSH had a capacity of 450–500 inpatient beds providing curative and preventive services for over 15 million people within the catchment area of approximately 15–16,000 km^2^. Although the hospital is rated as the local referral center, running water and hand sanitizer were not easily obtainable at the time of the study. Within 17 months, between January 2014 and June 2015 a total of 224 pure Gram-negative isolates were obtained from clinically apparent infections from routine clinical specimens in Jimma. Out of these, 14 could be identified as *Acinetobacter baumannii*. Three of these isolates were found to be meropenem resistant and NDM-1 positive. The samples originate from three independent inpatients (A–C) attending the surgical department of JUSH. Basic demographic and medical data were documented along with the specimens using standard clinical and laboratory record forms.

### Bacterial isolation and identification

The specimens were inoculated onto appropriate media according to standardized protocols. For isolation, 5% Columbia sheep blood agar as well as MacConkey II agar was used. Cultivation was performed in incubators at 37 °C for 24 and 48 h respectively. Blood agar plates were incubated at 5% CO_2_. After isolation, identification was performed by Gram-stain and using standardized biochemical reactions (API, bioMérieux SA, Marcy l’Etoile, France).

### Antimicrobial susceptibility testing

Isolates were tested against a common panel of antibiotics for their susceptibility pattern by disc diffusion method as outlined by Kirby-Bauer at JUSH bacteriology laboratory [18] and then retested for antibiotic susceptibilities at the Max von Pettenkofer-Institute (LMU) using disc-diffusion testing as well as VITEK 2 compact automated system (N215 and N248, bioMérieux, France) according to the manufacturer’s instructions. Minimal inhibitory concentrations (MICs) were determined by Etest (bioMérieux SA, Marcy l’Etoile, France) and interpreted according to the EUCAST guidelines [19].

### Molecular characterization

All strains were shipped to the Department of Bacteriology, Max von Pettenkofer-Institute (LMU), Munich, Germany for further investigation of their genetic traits using molecular methods. Upon receipt, all isolates were sub-cultured to ensure viability as well as purity and identified again to species level by MALDI-TOF mass spectrometry (MALDI Bioytper, Bruker Daltronic, Bremen, Germany) to exclude errors occurring during transport, labelling and reading the biochemical differentiation reactions. Bacterial DNA was isolated from a single colony using the High Pure PCR Template Preparation Kit (Roche Diagnostics, Mannheim, Germany) for genetic typing of resistance mechanisms and from 10 ml of stationary culture using the NucleoBond PC 20 (MACHEREY-NAGEL, Düren, Germany) kit as outlined by the manufacturers.

Genetic typing of resistance mechanisms was first performed as a screening with the Check-MDR CT102 Micro-Array Kit (Hain Lifescience, Nehren, Germany) following the manufacturer’s instructions, allowing for the detection of CTX-M ESBL enzyme subgroups, TEM Mutations, SHV Mutations, AmpC alleles CMY, DHA, FOX, MOX, ACC, MIR, ACT as well as Carbapenemases such as KPC, NDM-1, VIM, IMP, OXA-48-like. To also test for the carbapenemases common in *Acinetobacter spp.*, real time PCR reactions were performed to detect OXA-23-like, OXA24/40-like, OXA-51-like and OXA-58-like genes [20]. Whole genome sequencing was performed by Eurofins Genomics (Ebersberg, Germany). Multi Locus Sequence Typing (MLST) was performed *in silico* using the whole genome sequence data according to the Oxford-scheme [21] and the Sequence Types (ST) for each strain. The primers used for *in silico* MLST are detailed in the supporting information (see Additional file 1). The algorithm Neighbour-Joining was used to construct the phylogenetic tree with CLC Genomics Workbench 6.5 (CLC bio; Aarhus, Denmark). The *A. baumannii* sequences and the plasmid sequences used for comparison were received from the National Centre for Biotechnology Information (http://www.ncbi.nlm.nih.gov/). The strain information and the accession numbers are listed in the supporting information (see Additional file 2).

### Nucleotide accession numbers

The draft genomes of the three isolates A, B and C have been deposited at DDBJ/ENA/GenBank under the accession numbers LWSM00000000, LWSN00000000 and LWSO00000000 respectively.

## Results and discussion

In general, data on the distribution of antimicrobial resistance genes on the African continent is scarce, especially regarding carbapenemases. To our knowledge, no *bla*_NDM-1_-carrying bacteria or multidrug-resistant *A. baumannii* have been described in Ethiopia so far. In Kenya, an outbreak with NDM-1 producing *A. baumannii* was recently described, and strain types of the isolates have been determined [15]. Years before, *bla*_NDM-1_ could also be demonstrated in *Klebsiella* in neighbouring Kenya [22]. We detected three isolates of *A. baumannii* harbouring the *bla*_NDM-1_ gene within one hospital in Ethiopia, the Sequence Types were different than the isolates published from Kenya. We did not perform anal swab screening to explicitly search for *bla*_NDM-1_ positive bacteria; only clinically relevant materials were examined. However, the lack of other *bla*_NDM-1_-positive isolates is surprising, given the reports about the ease of horizontal transfer of NDM-1 containing plasmids [9, 23].

### The patients’ characteristics of the three NDM-1-carrying strains

Table 1 shows the basic demographic and medical data of the three patients. The hospital stay of patients’ A and C was at similar dates. Patients A and B recovered, the death of patient C was attributed to generalized infection with the NDM-1 expressing organism. Patient A suffered from hematopneumothorax and developed pleuritis growing high densities of pure *Acinetobacter baumannii* culture from putrid pleural fluid. She was treated with high dose trimethoprim/sulfamethoxazole after the antimicrobial susceptibility test result was available and improved with the therapy. This patient could be treated and survived the infection, whereas patient B and C did not offer any treatment option given the available drugs in JUSH. Patient C developed peritonitis after surgical intervention and grew predominantly *Acinetobacter baumannii* from peritoneal fluid. He died due to overwhelming systemic infection, whereas the otherwise young and healthy patient B growing pure *Acinetobacter baumannii* culture from an inguinal abscess was able to fight off the infection. During the course of infection, the process remained localized and treatment was given with antimicrobials which were tested resistant in the isolate.

**Table 1:** Basic demographic and medical data of patients A–C

Patient	A	B	C
Patient type	Inpatient	Inpatient	Inpatient
Department	ICU, surgical department	surgical department	ICU
Underlying disease	Hypertension	None	Asthma bronchiale
Clinical diagnosis	Hemopneumothorax	Inguinal abscess	Generalized peritonitis secondary to PUD and bronchial asthma, laparotomy
Specimen type	Fluid from hemopneumothorax	Abscess fluid/drain	Peritoneal swab
Antibiotics used during hospital stay	**cotrimoxazol**, ceftriaxone, metronidazol, ciprofloxacin, flucloxacillin	ceftazidime, cloxacillin, chloramphenicol	ceftriaxone, metronidazol, cloxacillin
Travel history	None	None	None
Outcome	Improved	Improved	Died

Treatment with substance tested susceptible depicted in bold

A summary of the antimicrobial susceptibility test results of the three *bla*_NDM-1_-carrying isolates can be seen in Table 2. One strain was found to differ regarding the susceptibility against trimethoprim/sulfamethoxazol (MIC 0.25 μg/ml vs. 32 μg/ml). Otherwise, the antimicrobial susceptibility patterns were identical, in all thre methods, VITEK automated testing, E-test as well as disc diffusion wherever applicable. Disc diffusion testing was performed on MH agar (Mueller-Hinton Agar, Becton Dickinson, Heidelberg, Germany) according to EUCAST v 4.0 criteria [19] and was read on an automated, camera based system (Adagio^TM^, Bio-Rad Laboratories GmbH, Munich, Germany). Growth of the three isolates was detected without any inhibition zone for chloramphenicol, ciprofloxacin, erythromycin, amoxicillin, cefoxitin, ceftazidime, cefepime and gentamicin. One isolate (B) showed an inhibition of 1 mm around the meropenem disc, the others did not. The raw values are detailed in Table 2. Of the substances with inhibition zones, EUCAST v 4.0 only offers cutoff values for disc diffusion for meropenem (S ≥ 21; R < 15 mm), ciprofloxacin (S ≥ 21; R < 21 mm), amikacin (S ≥ 18; R < 15 mm) and trimethoprim/sulfamethoxazole (S ≥ 16; R < 13 mm). The susceptibility determined with VITEK as well as E-test was confirmed by sufficient inhibition zones against amikacin (22, 23, 23 mm respectively) and trimethoprim/sulfamethoxazole for one isolate (25 mm). Remarkably, there was an inhibition zone around the tetracycline disc of 18, 20 and 21 mm respectively, which corresponds to a MIC determined by E-test of 12 μg/ml (Table 2). Despite the inhibition zone there cannot be any expected in vivo effectivity as tissue and serum levels even in high doses at peak concentrations should not exceed 5 μg/ml [24].

**Table 2:** Antimicrobial susceptibility testing results for the *Acinetobacter baumannii*

Antimicrobial compound	MIC breakpoint (μg/ml)		Isolate A		Isolate B		Isolate C	
			μg/ml	mm	μg/ml	mm	μg/ml	mm
	S ≤	R >	*A. baumannii*		*A. baumannii*		*A. baumannii*	
Chloramphenicol	–	–	>256	n.a.	>256	n.a.	>256	n.a.
Fosfomycin	–	–	>256	–	>256	–	>256	–
Ciprofloxacin	1	1	>32	n.a.	>32	n.a.	>32	n.a.
Erythromycin	–	–	>256	n.a.	>256	n.a.	>256	n.a.
Amoxicillin	–	–	>256	n.a.	>256	n.a.	>256	n.a.
Amoxicillin/Clavulanic Acid	–	–	>256	12	>256	13	>256	14
Cefoxitin	–	–	>256	n.a.	>256	n.a.	>256	n.a.
Ceftazidime	–	–	>256	n.a.	>256	n.a.	>256	n.a.
Cefepime	–	–	>256	n.a.	>256	n.a.	>256	n.a.
Meropenem	2	8	>32	n.a.	>32	8 **[21/15]**	>32	n.a.
Aztreonam	–	–	>256	–	>256	–	>256	–
Amikacin	8	16	**3**	**22 [18/15]**	**3.5**	**23 [18/15]**	**3.5**	**23 [18/15]**
Gentamicin	4	4	32	n.a.	32	n.a.	32	n.a.
Trimethoprim/Sulfamethoxazole	2	4	**0,25**	**25 [16/13]**	32	n.a.	32	n.a.
Polymyxin B	2	2	**1,5**	**13**	**1**	**17**	**1**	**17**
Tetracycline	–	–	12	18	12	20	12	21

strains Breakpoints and testing according to EUCAST v 4.0 [19]. Treatment options printed in bold. Disc diffusion diameters in mm in second column; n.a.: no inhibition around disc 6 mm); −:not performed; Disc diffusion diameter cutoff values shown in brackets in respective relevant fields, if no cutoff values are shown EUCAST v 4.0 does not indicate a cutoff value

To assess the transferability of the NDM-1 carrying genetic element to other *Enterobacteriaceae*, conjugation experiments against three different polymyxin B resistant *E. coli* mutant strains were conducted. Polymyxin B resistant mutants were chosen to allow for efficient selection of transformant *E. coli* strains, as the donor organism was resistant to almost all other substances. Conjugation was performed by co-cultivation of the two organisms in high density on solid Columbia 5% blood media (Becton Dickinson, Heidelberg, Germany) for different time periods, as well as in liquid media. Counter selection was performed with Polymyxin B. To enhance transformation efficiency, F+ strains were also employed. However, up to now, the *bla*_NDM-1_ gene could not be transferred to *E. coli*. This observation is obviously not sufficient to conclude that transfer might not occur between *Acinetobacter* strains or in accordance with the limited spread within the isolates encountered in Jimma. It might also be explained by low mobilization rates and chromosomal location of the gene. On the other hand, transposases were found *in silico* adjacent to the NDM-1-cassette and plasmid mobilization as well as pilus assembly genes showed up in the sequence data of the strain.

### Genetic traits and *Acinetobacter baumannii* phylogeny

**Fig. 1:**
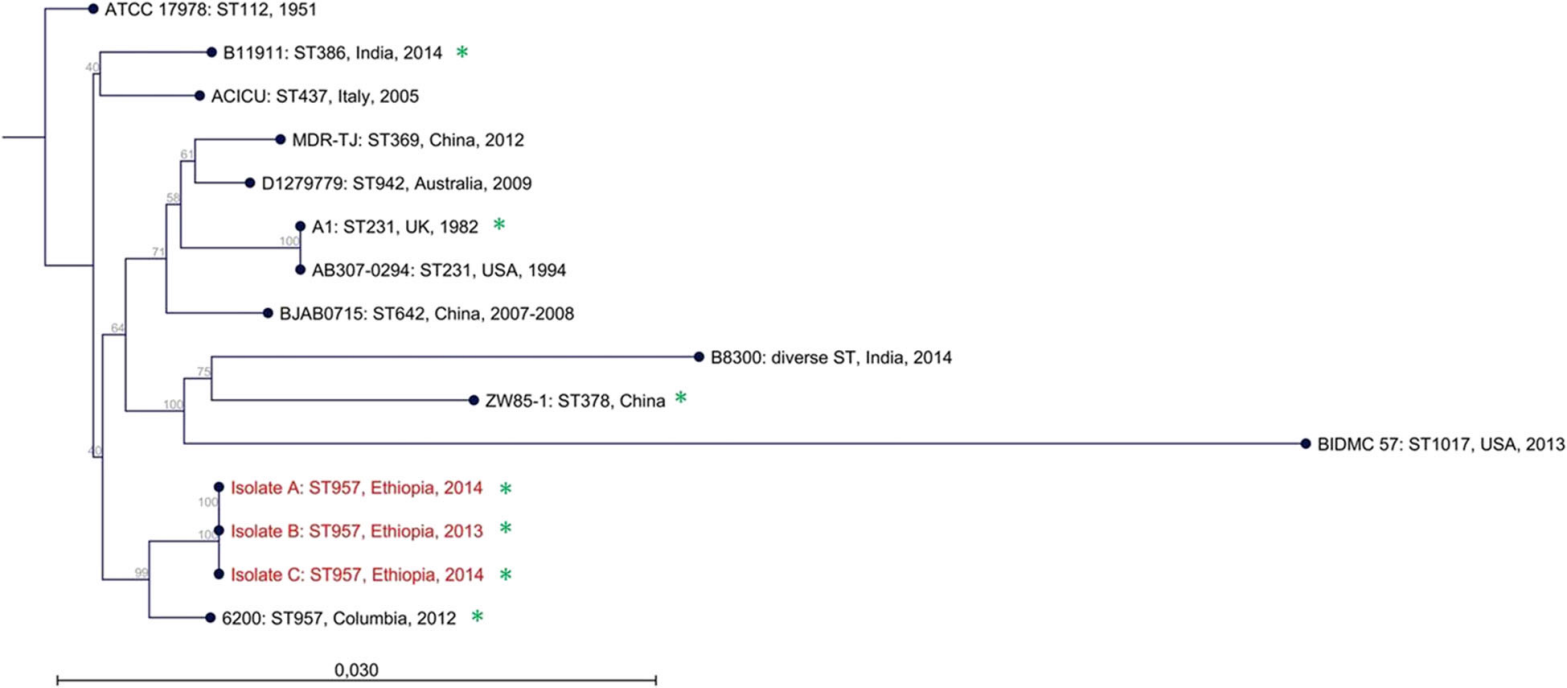
MLST-based phylogenetic tree using the Oxford-scheme. The Sequence Types, *countries* and *years* of isolation are indicated when known. *Green asterisks* indicate the proven presence of an NDM-1-plasmid (*Isolate A, Isolate B, Isolate C, 6200, B11911, A1, ZW85-1*). The *scale-bar* represents the number of SNPs per nucleotide

Upon sequencing, the three isolates exhibited an identical pattern regarding their MLST profile. It was identified to be ST957 (http://pubmlst.org/abaumannii/). Already published *A. baumannii* strains from Kenya had different STs indicating a different origin [15]. The nearest ancestor we could identify was strain 6200 isolated in Columbia in 2012 which was also grouped as ST957. While no single nucleotide polymorphism (SNP) was detected in the analysed housekeeping genes (concatenated sequence length: 4.284 bp) of the respective three isolates, the comparison to strain 6200 revealed 28 SNPs. Although a difference in trimethoprim/sulfamethoxazole resistance was found (Table 2), it is very possible, that these three isolates isolated in Jimma hospital from independent patients could reflect clonal diffusion. The phylogenetic tree for the documented genomes including isolates A, B and C is shown in Fig. 1. All three isolates also harboured the same 115 kb plasmid (without NDM-1) found in strain 6200, p6200 (NCBI accession number NZ_CP010398), corroborating the relationship to this strain.

**Fig. 2:**
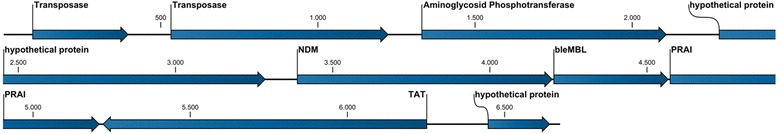
Gene-map of the NDM-1 cassette as sequenced in the three isolates from Ethiopia. The sequence was found to be identical to published sequences of NDM-1 in other organisms isolated from multiple places on different continents. There are clearly identifiable transposase genes as well as other antibiotic resistance genes in close proximity of NDM-1; PRAI: phosphoribosylanthranilate isomerase; TAT: twin-arginine translocation pathway

Unfortunately, we were unable to isolate or sequence the full length of the larger *bla*_NDM-1_ containing gene segment, most likely due to its vast size and repetitive sequence motifs. It is thus also possible, that the *bla*_NDM-1_ cassette was chromosomally encoded as has also been described in other strains and thus less easily transferred to other strains [25, 26]. The inner *bla*_NDM-1_ gene-cassette locus with several surrounding genes (about 6.6 kb) could be assembled from the sequences of all three strains and was highly similar to the *bla*_NDM-1_-cassettes of previously published strains. The *NDM*-*1* (813 bp) and the *bla*_MBL_-gene (366 bp) showed 100% sequence identity with the respective genes on the NDM-1-plasmids of the published *A. baumannii* strains as well as from *Acinetobacter lwoffi* strain Iz4b (NCBI accession number NC_025000) and *Providencia rettgeri* strain 09ACRGNY2001 (NCBI accession number KF295828). This again demonstrates the global distribution of the NDM-1 gene with vastly identical sequence. The genetic environment of *bla*_NDM-1_ is shown in Fig. 2.

## Conclusions

In this study, we describe *bla*_NDM-1_ gene carrying organisms from Ethiopia for the first time. The three *Acinetobacter baumannii* isolates from patients in Jimma were genetically analysed and were found to be distinct from the outbreak strains isolated in Kenya, the only characterized *bla*_NDM-1_ positive *Acinetobacter baumannii* strains in East Africa so far. This argues strongly against the regional spread of *bla*_NDM-1_ positive organism but rather suggests the independent, repetitive import of such strains from other regions. Clinicians should be aware of the possible emergence of outbreaks with different multi-resistant *Acinetobacter baumannii* strains. Although, no other bacterial species were found to harbour the gene cassette in Jimma so far, and conjugation was not successful under laboratory conditions, natural transmission to other bacteria seems likely given the evident lack of hygienic precautions due to limited resource settings. It is further likely, that the detected isolates are solely the tip of the iceberg regarding the presence of NDM-1 producing organisms in the region, as only a limited number of bacterial isolates could be evaluated. The three isolates were genetically very closely related and might represent clonal diffusion within the surgical department of the hospital.

## Additional files

Additional file 1: Primers used for in silico MLST. (DOCX 15 kb)
Additional file 2: Strain information and accession numbers. (DOCX 24 kb)

## Abbreviations

CHDL: Carbapenem-hydrolyzing class D ß-lactamases
CRAB: Carbapenem resistant *Acinetobacter baumannii*
ESBL: Extended-spectrum ß-lactamases
JUSH: Jimma University Specialized Hospital
MBL: Metallo ß-lactamases
MIC: Minimal inhibitory concentration
MLST: Multi locus sequence typing
NDM-1: New Delhi metallo-beta-lactamase
SNP: Single nucleotide polymorphism
ST: Sequence types
