# Supplementary material for: First report on blaNDM-1-producing Acinetobacter baumannii in three clinical isolates from Ethiopia
Source: BMC Infect Dis. 2017 Mar 1;17:180. doi: 10.1186/s12879-017-2289-9 (PMC5333390; doi:10.1186/s12879-017-2289-9)
Supplement: Additional file 1: — Primers used for in silico MLST. (DOCX 15 kb) [file 12879_2017_2289_MOESM1_ESM.docx]

| **Locus** | **Primer** | **Sequence** | **Amplicon size (bp)** |
| --- | --- | --- | --- |
| *gltA* | Citrato F1 | AAT TTA CAG TGG CAC ATT AGG TCC C | 722 |
|  | Citrato R12 | GCA GAG ATA CCA GCA GAG ATA CAC G |  |
| *gyrB* | gyrB_F | TGA AGG CGG CTT ATC TGA GT | 594 |
|  | gyrB_R | GCT GGG TCT TTT TCC TGA CA |  |
| *gdhB* | GDH SEC F | ACC ACA TGC TTT GTT ATG | 774 |
|  | GDH SEC R | GTT GGC GTA TGT TGT GC |  |
| *recA* | RA1 | CCT GAA TCT TCY GGT AAA AC | 425 |
|  | RA2 | GTT TCT GGG CTG CCA AAC ATT AC |  |
| *cpn60* | cpn60_F | GGT GCT CAA CTT GTT CGT GA | 640 |
|  | cpn60_R | CAC CGA AAC CAG GAG CTT TA |  |
| *gpi* | gpi_F | GAA ATT TCC GGA GCT CAC AA | 456 |
|  | gpi_R | TCA GGA GCA ATA CCC CAC TC |  |
| *rpoD* | rpoD-F | ACC CGT GAA GGT GAA ATC AG | 672 |
|  | rpoD-R | TTC AGC TGG AGC TTT AGC AAT |  |

**Table S1. Primers used for in silico MLST.**
