# Supplementary material for: First report on blaNDM-1-producing Acinetobacter baumannii in three clinical isolates from Ethiopia
Source: BMC Infect Dis. 2017 Mar 1;17:180. doi: 10.1186/s12879-017-2289-9 (PMC5333390; doi:10.1186/s12879-017-2289-9)
Supplement: Additional file 2: — Strain information and accession numbers. (DOCX 24 kb) [file 12879_2017_2289_MOESM2_ESM.docx]

| Strain | ST | Country | Year | Carbapenem resistance | Accession number | Reference |
| --- | --- | --- | --- | --- | --- | --- |
| **Isolate A** | 957 | Ethiopia | 2014 | NDM-1 | **LWSM00000000** | *This work* |
| **Isolate B** | 957 | Ethiopia | 2013 | NDM-1 | **LWSN00000000** | *This work* |
| **Isolate C** | 957 | Ethiopia | 2014 | NDM-1 | **LWSO00000000** | *This work* |
| 6200 | 957 | Columbia | 2012 | NDM-1 | NZ_CP010397.1 | Akers et al. |
| A1 | 231 | UK | 1982 | NDM-1 | CP010781.1 | Holt et al. |
| ZW85-1 | 378 | China | 2011 - 2013 | NDM-1 | NC_023028.1 | Wang et al. |
| D1279779 | 942 | Australia | 2009 | - | NC_020547.2 | Farrugia et al. |
| MDR-TJ | 369 | China | 2012 | + | NC_017847.1 | Huang et al. |
| BJAB0715 | 642 | China | 2007 - 2008 | + | CP003847.1 | Zhu et al. |
| AB307-0294 | 231 | USA | 1994 | - | CP001172.1 | Penwell et al. |
| ACICU | 437 | Italy | 2005 | + | CP000863.1 | Iacono et al. |
| ATCC 17978 | 112 |  | 1951 | - | CP000521.1 | Smith et al. |
| BIDMC 57 | 1017 | USA | 2013 | n.a. | NZ_KK737786.1 | Murphy et al. (direct submission) |
| B8300 | diverse | India | 2014 | n.a. | NZ_LFYY01000001.1 | Vijaykumar et al. (direct submission) |
| B11911 | 386 | India | 2014 | NDM-1 | NZ_LFYX01000003.1 | Balaji et al. |

**Table S2. Strain information and accession numbers.**

**References:**

Akers KS, Chaney C, Barsoumian A, Beckius M, Zera W, Yu X, et al. Aminoglycoside resistance and susceptibility testing errors in Acinetobacter baumannii-calcoaceticus complex. J Clin Microbiol. 2010 Apr;48(4):1132-8. doi: 10.1128/JCM.02006-09.

Balaji V, Rajenderan S, Anandan S, Biswas I. Genome Sequences of Two Multidrug-Resistant Acinetobacter baumannii Clinical Strains Isolated from Southern India. Genome Announc. 2015 Sep 10;3(5). pii: e01010-15. doi: 10.1128/genomeA.01010-15.

Farrugia DN, Elbourne LD, Hassan KA, Eijkelkamp BA, Tetu SG, Brown MH, et al. The complete genome and phenome of a community-acquired Acinetobacter baumannii. [PLoS One.](http://www.ncbi.nlm.nih.gov/pubmed/?term=The+Complete+Genome+and+Phenome+of+a+Community-+Acquired+Acinetobacter+baumannii) 2013;8(3):e58628. doi: 10.1371/journal.pone.0058628.

[Holt KE](http://www.ncbi.nlm.nih.gov/pubmed/?term=Holt%20KE%5BAuthor%5D&cauthor=true&cauthor_uid=25767221), [Hamidian M](http://www.ncbi.nlm.nih.gov/pubmed/?term=Hamidian%20M%5BAuthor%5D&cauthor=true&cauthor_uid=25767221), [Kenyon JJ](http://www.ncbi.nlm.nih.gov/pubmed/?term=Kenyon%20JJ%5BAuthor%5D&cauthor=true&cauthor_uid=25767221), [Wynn MT](http://www.ncbi.nlm.nih.gov/pubmed/?term=Wynn%20MT%5BAuthor%5D&cauthor=true&cauthor_uid=25767221), [Hawkey J](http://www.ncbi.nlm.nih.gov/pubmed/?term=Hawkey%20J%5BAuthor%5D&cauthor=true&cauthor_uid=25767221), [Pickard D](http://www.ncbi.nlm.nih.gov/pubmed/?term=Pickard%20D%5BAuthor%5D&cauthor=true&cauthor_uid=25767221), [Hall RM](http://www.ncbi.nlm.nih.gov/pubmed/?term=Hall%20RM%5BAuthor%5D&cauthor=true&cauthor_uid=25767221). Genome Sequence of Acinetobacter baumannii Strain A1, an Early Example of Antibiotic-Resistant Global Clone 1. [Genome Announc.](http://www.ncbi.nlm.nih.gov/pubmed/?term=Genome+Sequence+of+Acinetobacter+baumannii+Strain+A1%2C+an+Early+Example+of+Antibiotic-Resistant+Global+Clone+1) 2015 Mar 12;3(2). pii: e00032-15. doi: 10.1128/genomeA.00032-15.

[Huang H](http://www.ncbi.nlm.nih.gov/pubmed/?term=Huang%20H%5BAuthor%5D&cauthor=true&cauthor_uid=22952140), [Yang ZL](http://www.ncbi.nlm.nih.gov/pubmed/?term=Yang%20ZL%5BAuthor%5D&cauthor=true&cauthor_uid=22952140), [Wu XM](http://www.ncbi.nlm.nih.gov/pubmed/?term=Wu%20XM%5BAuthor%5D&cauthor=true&cauthor_uid=22952140), [Wang Y](http://www.ncbi.nlm.nih.gov/pubmed/?term=Wang%20Y%5BAuthor%5D&cauthor=true&cauthor_uid=22952140), [Liu YJ](http://www.ncbi.nlm.nih.gov/pubmed/?term=Liu%20YJ%5BAuthor%5D&cauthor=true&cauthor_uid=22952140), [Luo H](http://www.ncbi.nlm.nih.gov/pubmed/?term=Luo%20H%5BAuthor%5D&cauthor=true&cauthor_uid=22952140), et al. Complete genome sequence of Acinetobacter baumannii MDR-TJ and insights into its mechanism of antibiotic resistance. [J Antimicrob Chemother.](http://www.ncbi.nlm.nih.gov/pubmed/?term=Complete+genome+sequence+of+Acinetobacter+baumannii+MDR-TJ+and+insights+into+its+mechanism+of+antibiotic+resistance) 2012 Dec;67(12):2825-32. doi: 10.1093/jac/dks327.

[Iacono M](http://www.ncbi.nlm.nih.gov/pubmed/?term=Iacono%20M%5BAuthor%5D&cauthor=true&cauthor_uid=18411315), [Villa L](http://www.ncbi.nlm.nih.gov/pubmed/?term=Villa%20L%5BAuthor%5D&cauthor=true&cauthor_uid=18411315), [Fortini D](http://www.ncbi.nlm.nih.gov/pubmed/?term=Fortini%20D%5BAuthor%5D&cauthor=true&cauthor_uid=18411315), [Bordoni R](http://www.ncbi.nlm.nih.gov/pubmed/?term=Bordoni%20R%5BAuthor%5D&cauthor=true&cauthor_uid=18411315), [Imperi F](http://www.ncbi.nlm.nih.gov/pubmed/?term=Imperi%20F%5BAuthor%5D&cauthor=true&cauthor_uid=18411315), [Bonnal RJ](http://www.ncbi.nlm.nih.gov/pubmed/?term=Bonnal%20RJ%5BAuthor%5D&cauthor=true&cauthor_uid=18411315), et al. Whole-genome pyrosequencing of an epidemic multidrug-resistant Acinetobacter baumannii strain belonging to the European clone II group. [Antimicrob Agents Chemother.](http://www.ncbi.nlm.nih.gov/pubmed/?term=Whole-Genome+Pyrosequencing+of+an+Epidemic+Multidrug-Resistant+Acinetobacter+baumannii+Strain+Belonging+to+the+European+Clone+II+Grou) 2008 Jul;52(7):2616-25. doi: 10.1128/AAC.01643-07.

[Penwell WF](http://www.ncbi.nlm.nih.gov/pubmed/?term=Penwell%20WF%5BAuthor%5D&cauthor=true&cauthor_uid=22570720), [Arivett BA](http://www.ncbi.nlm.nih.gov/pubmed/?term=Arivett%20BA%5BAuthor%5D&cauthor=true&cauthor_uid=22570720), [Actis LA](http://www.ncbi.nlm.nih.gov/pubmed/?term=Actis%20LA%5BAuthor%5D&cauthor=true&cauthor_uid=22570720). The Acinetobacter baumannii entA gene located outside the acinetobactin cluster is critical for siderophore production, iron acquisition and virulence. [PLoS One.](http://www.ncbi.nlm.nih.gov/pubmed/?term=The+Acinetobacter+baumannii+entA+Gene+Located+Outside+the+Acinetobactin+Cluster+Is+Critical+for+Siderophore+Production%2C+Iron+Acquisition+and+Virulence) 2012;7(5):e36493. doi: 10.1371/journal.pone.0036493.

[Smith MG](http://www.ncbi.nlm.nih.gov/pubmed/?term=Smith%20MG%5BAuthor%5D&cauthor=true&cauthor_uid=17344419), [Gianoulis TA](http://www.ncbi.nlm.nih.gov/pubmed/?term=Gianoulis%20TA%5BAuthor%5D&cauthor=true&cauthor_uid=17344419), [Pukatzki S](http://www.ncbi.nlm.nih.gov/pubmed/?term=Pukatzki%20S%5BAuthor%5D&cauthor=true&cauthor_uid=17344419), [Mekalanos JJ](http://www.ncbi.nlm.nih.gov/pubmed/?term=Mekalanos%20JJ%5BAuthor%5D&cauthor=true&cauthor_uid=17344419), [Ornston LN](http://www.ncbi.nlm.nih.gov/pubmed/?term=Ornston%20LN%5BAuthor%5D&cauthor=true&cauthor_uid=17344419), [Gerstein M](http://www.ncbi.nlm.nih.gov/pubmed/?term=Gerstein%20M%5BAuthor%5D&cauthor=true&cauthor_uid=17344419), [Snyder M](http://www.ncbi.nlm.nih.gov/pubmed/?term=Snyder%20M%5BAuthor%5D&cauthor=true&cauthor_uid=17344419). New insights into Acinetobacter baumannii pathogenesis revealed by high-density pyrosequencing and transposon mutagenesis. [Genes Dev.](http://www.ncbi.nlm.nih.gov/pubmed/?term=New+insights+into+Acinetobacter+baumannii+pathogenesis+revealed+by+high-density+pyrosequencing+and+transposon+mutagenesis) 2007 Mar 1;21(5):601-14.

[Wang X](http://www.ncbi.nlm.nih.gov/pubmed/?term=Wang%20X%5BAuthor%5D&cauthor=true&cauthor_uid=24459253), [Zhang Z](http://www.ncbi.nlm.nih.gov/pubmed/?term=Zhang%20Z%5BAuthor%5D&cauthor=true&cauthor_uid=24459253), [Hao Q](http://www.ncbi.nlm.nih.gov/pubmed/?term=Hao%20Q%5BAuthor%5D&cauthor=true&cauthor_uid=24459253), [Wu J](http://www.ncbi.nlm.nih.gov/pubmed/?term=Wu%20J%5BAuthor%5D&cauthor=true&cauthor_uid=24459253), [Xiao J](http://www.ncbi.nlm.nih.gov/pubmed/?term=Xiao%20J%5BAuthor%5D&cauthor=true&cauthor_uid=24459253), [Jing H](http://www.ncbi.nlm.nih.gov/pubmed/?term=Jing%20H%5BAuthor%5D&cauthor=true&cauthor_uid=24459253). Complete Genome Sequence of Acinetobacter baumannii ZW85-1. [Genome Announc.](http://www.ncbi.nlm.nih.gov/pubmed/?term=Complete+Genome+Sequence+of+Acinetobacter+baumannii+ZW85-1) 2014 Jan 23;2(1). pii: e01083-13. doi: 10.1128/genomeA.01083-13.

[Zhu L](http://www.ncbi.nlm.nih.gov/pubmed/?term=Zhu%20L%5BAuthor%5D&cauthor=true&cauthor_uid=23826102), [Yan Z](http://www.ncbi.nlm.nih.gov/pubmed/?term=Yan%20Z%5BAuthor%5D&cauthor=true&cauthor_uid=23826102), [Zhang Z](http://www.ncbi.nlm.nih.gov/pubmed/?term=Zhang%20Z%5BAuthor%5D&cauthor=true&cauthor_uid=23826102), [Zhou Q](http://www.ncbi.nlm.nih.gov/pubmed/?term=Zhou%20Q%5BAuthor%5D&cauthor=true&cauthor_uid=23826102), [Zhou J](http://www.ncbi.nlm.nih.gov/pubmed/?term=Zhou%20J%5BAuthor%5D&cauthor=true&cauthor_uid=23826102), [Wakeland EK](http://www.ncbi.nlm.nih.gov/pubmed/?term=Wakeland%20EK%5BAuthor%5D&cauthor=true&cauthor_uid=23826102), et al. Complete genome analysis of three Acinetobacter baumannii clinical isolates in China for insight into the diversification of drug resistance elements. [PLoS One.](http://www.ncbi.nlm.nih.gov/pubmed/?term=Complete+Genome+Analysis+of+Three+Acinetobacter+baumannii+Clinical+Isolates+in+China+for+Insight+into+the+Diversification+of+Drug+Resistance+Elements) 2013 Jun 24;8(6):e66584. doi: 10.1371/journal.pone.0066584.
